# Overexpression of human wild-type FUS causes progressive motor neuron degeneration in an age- and dose-dependent fashion

**DOI:** 10.1007/s00401-012-1043-z

**Published:** 2012-09-09

**Authors:** Jacqueline C. Mitchell, Philip McGoldrick, Caroline Vance, Tibor Hortobagyi, Jemeen Sreedharan, Boris Rogelj, Elizabeth L. Tudor, Bradley N. Smith, Christian Klasen, Christopher C. J. Miller, Jonathan D. Cooper, Linda Greensmith, Christopher E. Shaw

**Affiliations:** 1 Department of Clinical Neuroscience, Kings College London, King’s Centre for Neurodegeneration Research, London, SE5 8AF UK; 2 Sobell Department of Motor Neuroscience and Movement Disorders, MRC Centre for Neuromuscular Disease, UCL Institute of Neurology, Queen Square, London, WC1N 3BG UK; 3 Department of Neuroscience, Institute of Psychiatry, London, SE5 8AF UK; 4 Genetics of Metabolic and Reproductive Disorders, Max Delbrück Center for Molecular Medicine, Robert-Rössle-Str, 10, 13215 Berlin, Germany

## Abstract

Amyotrophic lateral sclerosis (ALS) and frontotemporal lobar degeneration (FTLD) are relentlessly progressive neurodegenerative disorders with overlapping clinical, genetic and pathological features. Cytoplasmic inclusions of fused in sarcoma (FUS) are the hallmark of several forms of FTLD and ALS patients with mutations in the *FUS* gene. FUS is a multifunctional, predominantly nuclear, DNA and RNA binding protein. Here, we report that transgenic mice overexpressing wild-type human FUS develop an aggressive phenotype with an early onset tremor followed by progressive hind limb paralysis and death by 12 weeks in homozygous animals. Large motor neurons were lost from the spinal cord accompanied by neurophysiological evidence of denervation and focal muscle atrophy. Surviving motor neurons in the spinal cord had greatly increased cytoplasmic expression of FUS, with globular and skein-like FUS-positive and ubiquitin-negative inclusions associated with astroglial and microglial reactivity. Cytoplasmic FUS inclusions were also detected in the brain of transgenic mice without apparent neuronal loss and little astroglial or microglial activation. Hemizygous FUS overexpressing mice showed no evidence of a motor phenotype or pathology. These findings recapitulate several pathological features seen in human ALS and FTLD patients, and suggest that overexpression of wild-type FUS in vulnerable neurons may be one of the root causes of disease. Furthermore, these mice will provide a new model to study disease mechanism, and test therapies.

**Electronic supplementary material:**

The online version of this article (doi:10.1007/s00401-012-1043-z) contains supplementary material, which is available to authorized users.

## Introduction

Amyotrophic lateral sclerosis (ALS) and frontotemporal lobar degeneration (FTLD) are relentlessly progressive neurodegenerative disorders. ALS is characterised by the degeneration of motor neurons in the motor cortex and spinal cord, progressive paralysis and ultimately death due to respiratory failure [9, 46]. FTLD is the second most common cause of dementia with an onset before 65 years. It is characterised by focal atrophy of the frontal and/or temporal lobes, causing changes in personality, behaviour and language [52]. ALS and FTLD are increasingly recognised as the phenotypic ends of a clinical spectrum as ~15 % of patients with ALS have cognitive and language deficits akin to FTLD [47]. Conversely, around 15 % of people with FTLD develop clinical signs of ALS [32, 40].

ALS and FTLD also have overlapping molecular pathology [31] as ~95 % of ALS cases and ~50 % of FTLD have neuronal cytoplasmic inclusions (NCIs) containing the TAR DNA binding protein (TDP-43) [35, 45]. TDP-43 binds to a large number of RNA targets and is involved in the regulation of transcription, splicing and trafficking [51]. In a minority of ALS and/or FTLD cases, cytoplasmic aggregation of TDP-43 and its loss from the nucleus is associated with pathogenic mutations in the genes encoding TDP-43 (*TARDBP*[50]), progranulin (*PGRN* [33]), valosin containing protein (*VCP*[42]) and Ubiquilin 2 (*UBQLN2* [10]).

Mutations in another RNA binding protein, fused in sarcoma (FUS), have been identified in ~4 % of autosomal dominant familial ALS [28, 55] and in rare cases of FTLD [54]. FUS is ubiquitously expressed and plays an important role in the regulation of RNA transcription, splicing and transport [4, 15, 58]. Although predominantly nuclear in distribution under physiological conditions, in patients with pathogenic mutations FUS protein accumulates in inclusions in the cytoplasm of lower motor neurons in the spinal cord [11, 28, 34, 55]. Unlike many other neurodegenerative diseases, mutant FUS aggregates in ALS are not decorated by ubiquitin or p62 [55]. The majority of FUS mutations occur in the extreme C-terminus of the protein, which contains a nuclear localisation signal [13], thus it has been proposed that either loss of protein from the nucleus, or toxicity of protein aggregates accumulated in the cytoplasm leads to neurodegeneration [12, 39].

Cytoplasmic and nuclear FUS inclusions have also been identified as the dominant protein deposited in FTLD cases previously classified as neuronal intermediate filament inclusion disease (NIFID), basophilic inclusion body disease (BIBD) and atypical FTLD with ubiquitinated inclusions [38, 43, 44, 53]. In contrast to ALS cases, FUS mutations in FTLD are rare and the pathology is distinct. The RNA binding proteins TATA-binding protein-associated factor (TAF15) and Ewings Sarcoma protein (EWS) colocalise with FUS within inclusions in FTLD, which may explain why these inclusions appear to be ubiquitinated.

Knockout of Fus in inbred strains of mice results in chromosomal instability and perinatal death [20], but only causes male sterility and enhanced sensitivity to radiation in outbred animals [27]. Neither Fus−/− lines are reported to show motor or cortical neuronal loss. Overexpression of human ALS mutant FUS in adult transgenic rats, however, results in progressive paralysis secondary to the degeneration of motor axons and substantial loss of cortical and hippocampal neurons [21]. Overexpression of the wild-type protein resulted in cognitive deficits in older animals due to a loss of cortical and hippocampal neurons [21]. This indicated that mutant FUS is more toxic to lower motor neurons than normal FUS, but that increased expression of wild-type FUS is sufficient to cause neurodegeneration.

Here we report the effect of wild-type FUS overexpression in inbred mice, and demonstrate significant loss of motor neurons, coupled with a major motor and pathological phenotype that recapitulates many aspects of FUS-ALS. This phenotype appears to be crucially dependent on the expression level of the protein, and is associated with a significant shift in FUS localisation to the cytoplasm without concomitant loss of FUS from the nucleus.

## Materials and methods

### Ethics statement

All experiments were performed under the terms of the UK Animals (Scientific Procedures) Act 1986, and were approved by the Kings College, London ethics review panel.

### Transgenic animals

For creation of transgenic mice, HA-tagged human FUS cDNA was cloned into a modified mouse prion gene and following removal of vector sequences, C57Bl/6/SJL founder mice were produced by pronuclear injection as described [30]. Animals were backcrossed onto C57Bl/6 three times prior to further breeding, following which hemizygous animals were interbred to produce homozygous offspring. Direct sequencing of the FUS transgene was performed with Big-Dye^®^ Terminator v1.1 on an ABI3130 genetic analyser (Applied Biosystems). All sequence traces were analysed using Sequencher^®^ 4.10 (Gene Codes Corporation). Hemizygous animals were identified using PCR with primers 5′-GCAGGGCTATTCCCAGCAGAGCAG and 3′-CTGGTTCTGCTGTCCATAGCCCTG. Homozygous mice were identified using qPCR with primers 5′-CAGCAAAGCTATGGACAGC and 3′-GCGGTTATGGCAATCAAGAC and a FAM/TAMRA tagged probe-AGCAGAACCAGTACAACAGCAGCA. GAPDH was used as a housekeeping control. Each sample was measured in triplicate. hFUS (+/−) mice will be available through the Jackson Laboratory Repository. They are assigned JAX stock no. 017916.

### Evaluation of motor function and health

From 3 weeks of age, NTg (*n* = 9), hFUS (+/−) (*n* = 17) and hFUS (+/+) (*n* = 11) mice were weighed on a weekly basis, and general health status was recorded. Animals showing signs of hind limb paralysis were monitored daily, and disease end-stage and death was defined as the time when animals could no longer obtain food or water, or had lost 25 % of their body weight, at which point they were euthanized.

Motor strength and coordination were longitudinally evaluated on the rotarod (Columbus Instruments), using a 5-min accelerating protocol starting at 2 rpm, and rising to 30 rpm throughout the 5-min testing period. Mice were tested once a week, and latency to fall was recorded. All mice received an initial training session of 2 min at 2 rpm to acclimatise them to the equipment. Data were assessed statistically by two-way analysis of variance (ANOVA) followed by the post hoc Holm-Sidak test. At eight weeks of age, the locomotor activity of animals was assessed in an 80-cm diameter circular open field environment. Mice were allowed to explore the open field for 10 min. Trials were monitored and analyzed using the Ethovision package (Noldus, The Netherlands), and total distance travelled was recorded. Data were assessed statistically by way of one-way ANOVA followed by the post hoc Tukey test.

### Histology and immunohistochemistry

Eleven-week-old, end-stage mice, and their age-matched littermates were anaesthetised and transcardially perfused with PBS, followed by 4 % paraformaldehyde (PFA) in phosphate buffer. Brain and spinal cords were postfixed in 4 % PFA in 15 % sucrose for 5 h, cryoprotected in 30 % sucrose for 24 h and cut into 30 μm sections on a cryostat.

For immunohistochemistry, the following antibodies were used: rabbit anti-FUS (1:500, Sigma), rabbit anti-ubiquitin (1:1000, DAKO), rat anti-HA (1:5000, Roche), rabbit anti-GFAP (1:4000, DAKO), mouse anti-CD68 (1:2000, Serotec), goat anti-EWS (C-19; 1:50, Santa Cruz) and TAF15 (TAF II p68; 1:50, Santa Cruz). Sections were washed and incubated with the appropriate biotinylated secondary antibody (1:1000, Vector), and then with an ABC kit (Vector). Sections were imaged using a Zeiss light or confocal microscope and axiovision software.

For motor neuron counts, perfused lumbar spinal cords from four animals per genotype were serially sectioned, and every 6th section (30 μm) was analysed. Sections were mounted, dried, incubated overnight in 1:1 ethanol/chloroform to de-fat the sections, stained for 10 min in warm 0.1 % cresyl violet, dehydrated and coverslipped. To compare the number of motor neurons, large neurons greater than 30 μm in diameter (based on their Fret’s diameter as assessed by Image J software) in the anterior horn of the lumbar spinal cord were counted in 15 sections. Data were assessed statistically by one-way ANOVA, followed by the post hoc Tukey test.

For muscle histology, gastrocnemius or tibialis anterior muscles were dissected fresh, immediately frozen in isopentane cooled in dry ice, and cryostat sections were cut onto slides and stained with haematoxylin and eosin or succinate dehydrogenase (SDH) activity to determine oxidative capacity of the muscle fibres, as described [52].

### Immunoblotting

For FUS and HA expression level analysis, whole brains of four end-stage hFUS (+/+) and four age-matched hFUS (+/−) and NTg animals were lysed in low salt buffer (10 mM Tris, 5 mM EDTA, 10 % sucrose) with protease inhibitors (Roche Diagonstics). For cytoplasmic and nuclear fractionation, four brain samples for each genotype were prepared as described earlier [19]. Briefly, snap-frozen tissue was weighed and homogenised in buffer containing 10 mM Hepes, 10 mM NaCl, 1 mM KH_2_PO_4_, 5 mM NaHCO_3_, 5 mM EDTA, 1 mM CaCl_2_, 0.5 mM MgCl_2_ and protease inhibitors (10× vol/weight). After 10 min on ice, 2.5 M sucrose (0.5× vol/weight) was added. Tissue was homogenized and centrifuged at 6,300*g* for 10 min. The supernatant was collected as the cytoplasmic fraction. The pellet was washed 4 times in TSE buffer [10 mM Tris, 300 mM sucrose, 1 mM EDTA, 0.1 % IGEPAL (Sigma) and protease inhibitors 10× vol/weight], homogenized and centrifuged at 4,000×*g* for 5 min. Finally, the pellet was resuspended in RIPA buffer with 2 % SDS (5 × vol/weight) as the nuclear fraction. Protein samples were then separated by SDS/PAGE using 10 % polyacrylamide gels, and transferred to nitrocellulose membranes. Total FUS was recognised by a rabbit polyclonal antibody to FUS (1:2,000, Novus Biologicals), and exogenous HA tagged human FUS was recognised by a mouse monoclonal antibody to the HA tag (1:1,000, Cell Signalling). Fluorescent secondary antibodies conjugated to Dylight 680 or 800 nm (Thermo Scientific) were used to detect protein levels, and results were visualised using the Odyssey Imager (Licor). Data were normalised to GAPDH (1:5,000, Sigma) or Lamin B1 (1:2,000, Abcam). Quantitation of immunoblots was done using Image J software, and data were analysed statistically by way of ANOVA followed by the post hoc Tukey test.

### In vivo physiological assessment of neuromuscular function and motor unit survival

Functional analysis of hind limb muscle function was undertaken at 70 days. NTg (*n* = 5), hFUS(+/−) (*n* = 6) and hFUS(+/+) (*n* = 6) mice were anaesthetised (4.5 % chloral hydrate, 1 ml/100 g of bodyweight, i.p) and prepared for in vivo analysis of isometric muscle force as previously described [24]. The distal tendons of the tibialis anterior (TA) and extensor digitorum longus (EDL) hind limb muscles were dissected free and attached by silk thread to isometric force transducers (Dynamometer UFI Devices, Welwyn Garden City, UK). The sciatic nerve was exposed and sectioned proximally. The length of the muscles was adjusted for maximum twitch tension. The muscles and nerves were kept moist with saline through recordings and all experiments were carried out at room temperature. Isometric contractions were elicited by stimulating the nerve to the TA and EDL using square wave pulses of 0.02 ms duration at supra-maximal intensity, via silver wire electrodes. Contractions were elicited by either a single stimulus for twitch tension or trains of stimuli at frequencies of 40, 80 and 100 Hz for tetanic tension. The maximum twitch and tetanic tension was measured using a computer and Picoscope software (Pico Technology, Cambridgeshire, UK).

Following recording of isometric tension, the contractile and fatigue characteristics of EDL muscles were determined. The time to peak (TTP) was calculated by measuring the time taken (ms) for the muscle to elicit peak twitch tension and the half relaxation time (the time taken for the muscle to reach half relaxation from peak contraction) was also calculated. In addition, the resistance of the EDL muscles to fatigue was assessed by repeated stimulation at 40 Hz for 250 ms every second for 3 min. The tetanic contractions were recorded on a Lectromed Multitrace 2 recorder (Lectromed Ltd, UK). The decrease in tension after 3 min of stimulation was measured and a fatigue index (FI) was calculated, where a FI approaching a value of 1.0 indicating that a muscle is very fatigable [52].

The number of motor units innervating the EDL muscles was determined by stimulating the nerve with stimuli of increasing intensity, resulting in stepwise increments in twitch tension due to successive recruitment of motor axons with increasing stimulus thresholds. The number of stepwise increments was counted to give an estimate of the number of functional motor units in the EDL muscles [52].

## Results

### Overexpression of human wild-type FUS causes progressive paralysis and death in mice

**Fig. 1:**
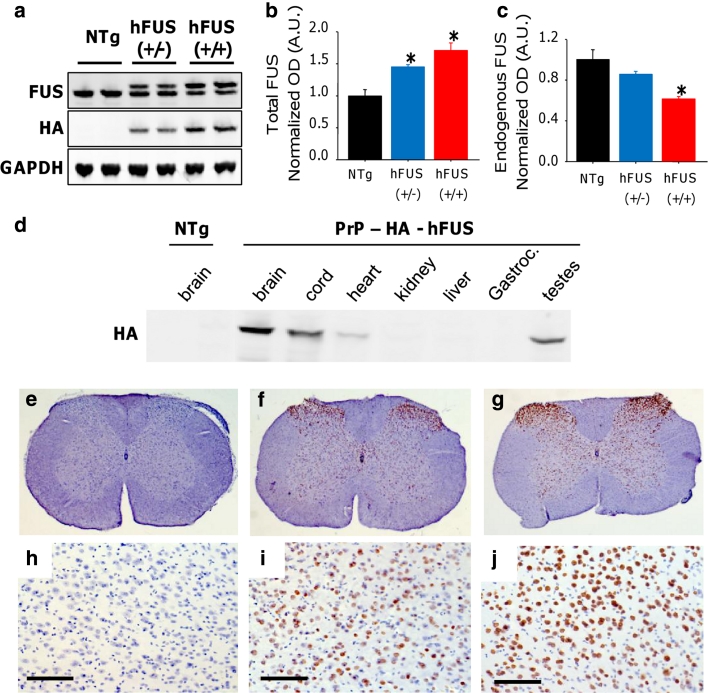
Overexpression of hFUS in mice dose dependently decreases endogenous Fus expression. **a** Western blotting of brain lysate from NTg and hFUS (+/−) and (+/+) mice using an anti-FUS antibody showed a slight shift to a higher molecular weight due to the presence of the HA tag and the larger protein size, and there was a dose-dependent increase in HA-hFUS and total FUS expression in transgenic animals (**b**), accompanied by a concomitant decrease in endogenous Fus expression (**c**) (mean ± SEM; * *p* < 0.05). **d** Western blotting of tissue lysates with anti-HA antibody showed highest levels in brain, spinal cord and testis, with lower levels in skeletal muscle (*Gastroc* gastrocnemius), heart and other tissues. (**e**–**j**) Immunohistochemistry with anti-HA antibody showed expression of the hFUS protein throughout the spinal cord (**e**–**g**) and in the cortex (**h**–**j**; *scale bar* 100 μm) in both hFUS (+/−) (**f**, **i**) and (+/+) (**g**, **j**) mice, which was absent in NTg animals (**e**, **h**)

From 33 potential founders, only 3 were shown to carry the human FUS (hFUS) construct, with an N-terminal HA-tag, and under the control of the mouse prion protein (PrP) promoter. The presence of the human wild-type *FUS* transgene was confirmed using direct sequencing in all three founders. Although all three founders bred, only one founder generated F1 mice that were transgenic for *FUS* and could be bred forward onto a C57B6 background. Difficulties in producing constitutively expressing transgenic animals appear to be a feature of both Fus and TDP-43 models, with other reports featuring a single line of mice [56]. Western blots of brain lysates from the PrP-hFUS F3 generation mice showed that the exogenous hFUS protein has a slightly higher molecular weight than endogenous mouse FUS due to eight additional amino acids and the presence of the HA-tag (Fig. 1a). The HA-hFUS expression profile of major organs showed that the PrP-hFUS transgene was expressed highest in the brain, spinal cord and testis, with much lower expression in other tissues, which is typical for gene expression driven-PrP promoter [5] (Fig. 1d). Quantification of expression indicated that total FUS expression detected in the brain of hemizygous animals [hFUS (+/−)] was only 1.4-fold higher than non-transgenic (NTg) animals (Fig. 1a, b). In order to increase transgene dose, we generated “homozygous” mice [hFUS (+/+)], which have a 1.9-fold increase in HA-hFUS expression compared to their hFUS (+/−) littermates (Fig. 1a). This increase however was associated with a down-regulation of endogenous murine Fus in transgenic animals (Fig. 1a, c), with levels 0.86 [hFUS (+/−)] and 0.62 [hFUS (+/+)] fold lower than NTg animals. Thus, hFUS (+/+) mice have only a 1.7-fold increase in total FUS levels compared to their NTg littermates (Fig. 1a, b). Immunohistochemistry using a HA-tag antibody to selectively visualise the exogenous hFUS protein showed expression throughout the spinal cord (Fig. 1e–g), and in the cortex (Fig. 1h–j) in both (+/−) and (+/+) hFUS mice.

**Fig. 2:**
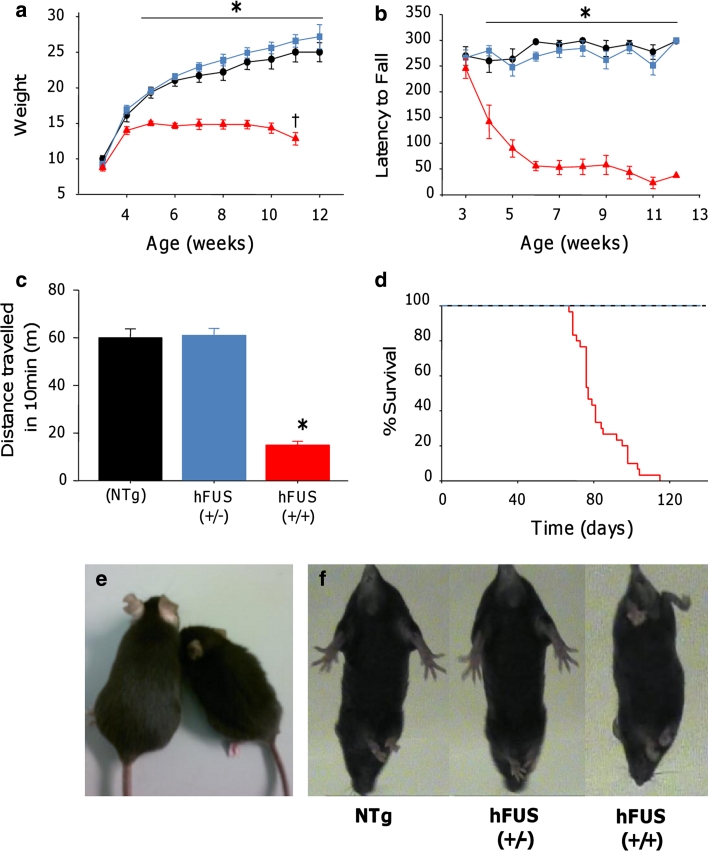
Overexpression of hFUS in mice results in motor dysfunction and premature death. **a** hFUS (+/+) (*filled triangles*) mice show a failure to gain weight from 4 weeks of age, and are significantly lighter than their NTg (*filled circle*) and hFUS (+/−) (*filled rectangle*) littermates from 5 weeks old mice. In addition, they display a significant reduction in weight from their maximum at 11 weeks of age. **b** hFUS (+/+) mice display significant impairment and rapid decline in performance on the rotarod from 4 weeks of age. **c** 8-week-old hFUS (+/+) mice display a significant reduction in locomotor activity compared to their NTg and hFUS (+/−) littermates. All data shown are mean ± SEM; * *p* < 0.05. **d** Survival curve showing an average survival of 82 ± 12 days for hFUS (+/+) mice, while hFUS (+/−) survival does not significantly differ from NTg mice. **e** Representative example of an 8-week-old hFUS (+/+) mice (*right animal*) compared to its NTg littermate (*left animal*). **f** Abnormal hind limb splay in 8-week-old hFUS (+/+) mice

Weight, general health and motor function, using the accelerating rotarod paradigm, were assessed on a weekly basis from three weeks of age. Animals were born at Mendelian ratios, and were indistinguishable from their NTg littermates until 4 weeks of age when the (+/+) animals began to develop a tremor (Online resource Movie S1) and show mild signs of hind limb dysfunction characterised by a slightly stilted gait (Online resource Movie S2). From 4 weeks, hFUS (+/+) mice displayed a failure to gain weight normally (Fig. 2a, e) and a rapid decline in their motor function, displaying a significant reduction in performance on the accelerating rotarod compared with NTg and hFUS (+/−) littermates (Fig. 2b). By 8 weeks of age, hFUS (+/+) mice showed a significant reduction in general locomotor activity in an open field environment compared to their NTg and hFUS (+/−) littermates (Fig. 2c). They displayed signs of progressive hind limb paralysis with a severely stilted gait, an inability to raise their pelvis off the ground (Online resource Movie S3) and a failure to splay hind limbs normally when lifted by their tail (Fig. 2f). Following the onset of hind limb paralysis at 7–8 weeks, hFUS (+/+) mice displayed signs of rapid disease progression, and were euthanized by 10–13 weeks because they were unable to obtain food or water, or had lost 25 % of their total body weight. The average survival time for hFUS (+/+) mice was 82 ± 12 days (Fig. 2d). In contrast, hFUS (+/−) animals gained weight normally, displayed no significant motor dysfunction or signs of ill health, and appear to have a life span comparable with their NTg littermates (at least 104 weeks to date).

### Motor dysfunction and death is accompanied by an increase in cytoplasmic FUS but no ubiquitinated FTLD-FUS-like inclusions

The pathological hallmarks of FTLD-FUS cases are ubiquitin-positive cytoplasmic inclusions containing FUS in neurons within the frontal and temporal lobes [43, 48]. In contrast, ALS-FUS cases are characterised by ubiquitin-negative FUS-positive inclusions within motor neurons in the spinal cord and brain stem [55]. We therefore examined the localisation of FUS and ubiquitin in the brains and spinal cords of hFUS mice using immunohistochemistry.

**Fig. 3:**
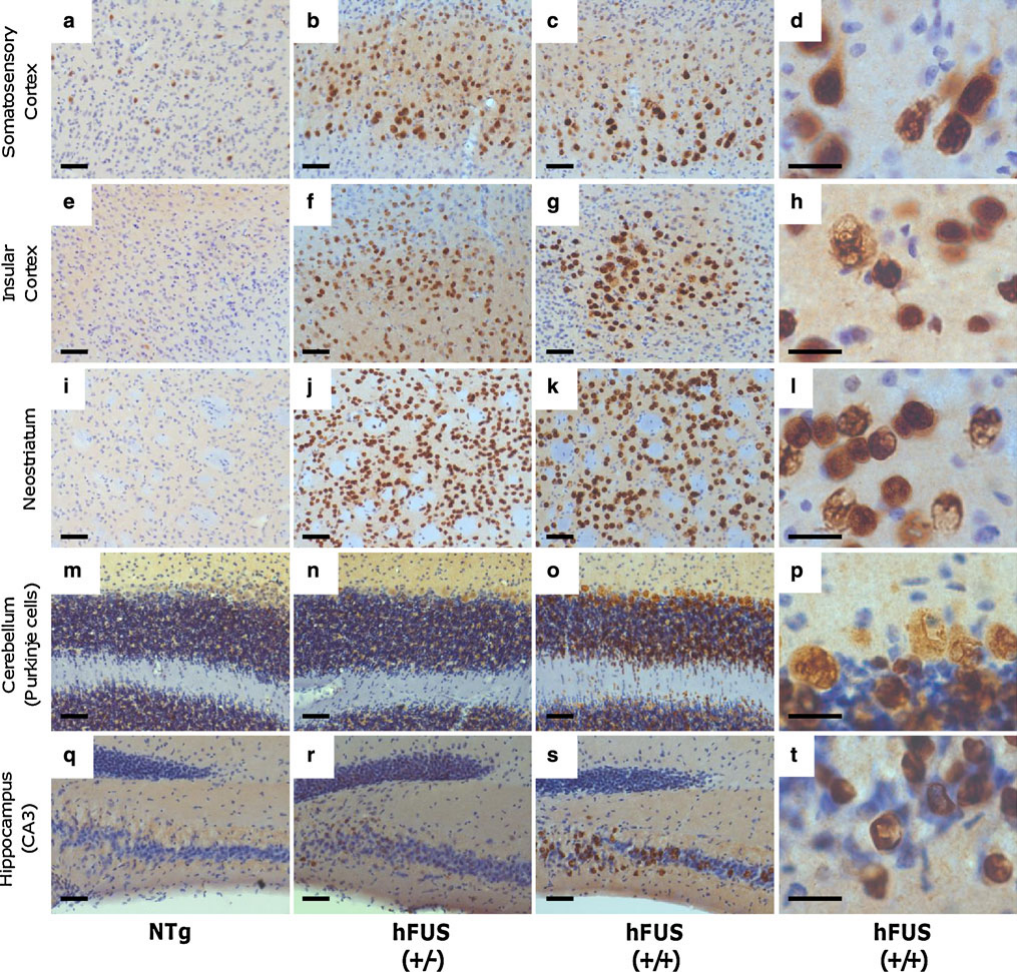
FUS staining in brain regions associated with motor or cognitive function in hFUS animals. Overexpression of HA-hFUS resulted in a dose-dependent increase in FUS staining in the somatosensory (**a**–**c**) and insular (**e**–**g**) cortices, the neostriatum (**i**–**k**), the Purkinje cells of the cerebellum (**m**–**o**) and a portion of the CA3 region of the hippocampus (**q**-**s**) (*scale bar* 50 μm). Higher power images of homozygous animals reveal skein-like and diffuse cytoplasmic staining in the somatosensory (**d**) and insular (**h**) cortices, the neostriatum (**i**) and the Purkinje cells of the cerebellum (**p**) and to a much lesser extent in the hippocampus (**t**) (*scale bar* 20 μm)

**Fig. 4:**
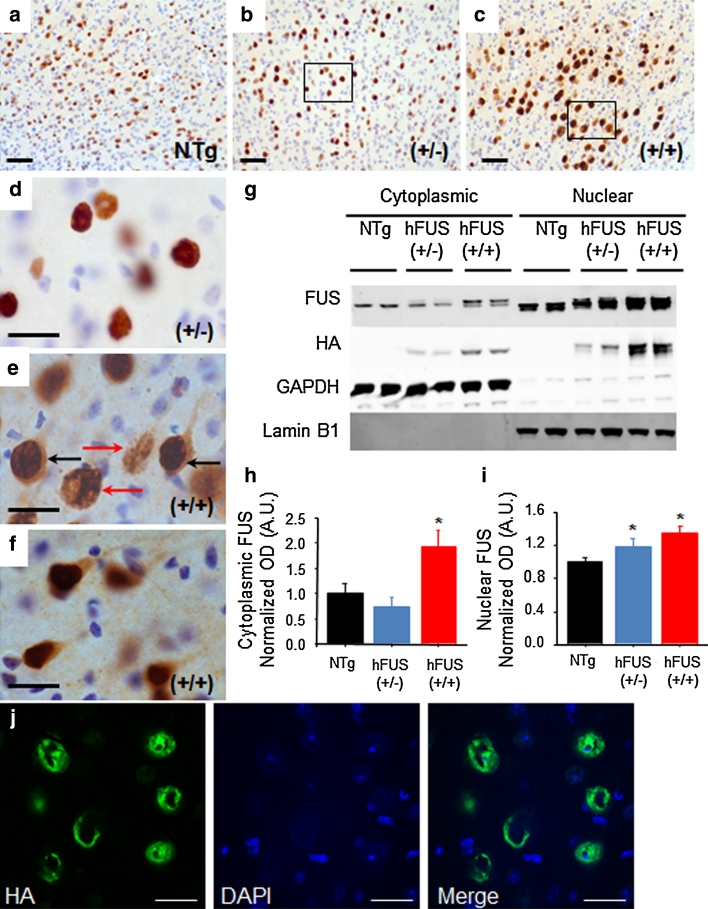
hFUS overexpression results in a significant shift in cytoplasmic localisation in homozygous animals. (**a**–**c**) FUS expression in the motor cortex was increased in both (+/−) (**b**) and (+/+) (**c**) hFUS mice compared to their NTg (**a**) littermates (*scale bar* 50 μm). **d** A higher power image of the hFUS (+/−) section reveals mainly nuclear and perinuclear FUS staining (*scale bar* 20 μm). **e** A higher power image of the hFUS (+/+) section shows many neurons with cytoplasmic staining (*black arrows*) that is absent in both hFUS (+/−) and NTg mice. In addition, ring-like perinuclear FUS staining is evident in numerous cells in both hFUS (+/+) (*red arrows*), and to a lesser extent hFUS (+/−) animals (*scale bar* 20 μm). **f** A second high power image showing cytoplasmic staining in cortical neurons of the hHUS (+/+) brain even in the absence of abnormal ring-like accumulations. **g** Western blotting of nuclear and cytosolic fractions from brain demonstrated an increase in both cytosolic and nuclear FUS levels. GAPDH was used as the marker for the cytosolic fraction, and Lamin B1 for the nuclear fraction. **h** Quantification of cytosolic FUS levels, showing an increase in FUS levels only in hFUS (+/+) animals (* *p* < 0.05). **i** Quantification of nuclear FUS levels, showing an expression-dependent increase in FUS in transgenic mice (* *p* < 0.05). **j** A representative confocal image demonstrating the ring-like nature of the inclusions observed in hFUS (+/+) cortical neurons (*scale bar* 20 μm)

Intense FUS staining, with numerous ring-like perinuclear inclusions (Fig. 4j), was observed in end-stage hFUS (+/+) mice, and to a lesser extent in age-matched hFUS (+/−) animals, in layer V neurons of the motor and somatosensory cortices, the insular cortex, the neostriatum and the Purkinje cells of the cerebellum (Figs. 3, 4). Intense staining was also seen in a subset of pyramidal cells in the CA3 region of the hippocampus (Fig. 3). In general, FUS immunoreactivity in hFUS (+/−) mice was confined predominantly to the nuclear and perinuclear area of neurons (Fig. 4b, d), with a distribution similar to that seen in NTg animals (Fig. 4a). In contrast, hFUS (+/+) mice also displayed diffuse FUS staining throughout the cytoplasm of cortical neurons (Fig. 4c, e, f) with no apparent loss of nuclear FUS.

FUS, identified as a predominantly nuclear protein, accumulates and aggregates in the cytoplasm in both FUS-ALS and FUS-FTLD [43, 55] and the apparent increase in cytoplasmic staining in hFUS (+/+) mice suggests that FUS overexpression in these animals may significantly increase cytoplasmic FUS levels. To further investigate this possibility, FUS levels in nuclear and cytoplasmic fractions prepared from brain samples were analysed by immunoblotting. In the brain, hFUS (+/−) and (+/+) animals show a significant, and dose-dependent increase in nuclear FUS levels compared to NTg controls (*p* = 0.044 and 0.006, respectively; Fig. 4g, i), in contrast, cytoplasmic FUS levels were unchanged in hFUS (+/−) animals (*p* = 0.593), but rose dramatically in hFUS (+/+) mice (*p* = 0.002; Fig. 4g, h). Thus, localisation of FUS to the cytosol appears to be critically dependent on the level of FUS expression.

**Fig. 5:**
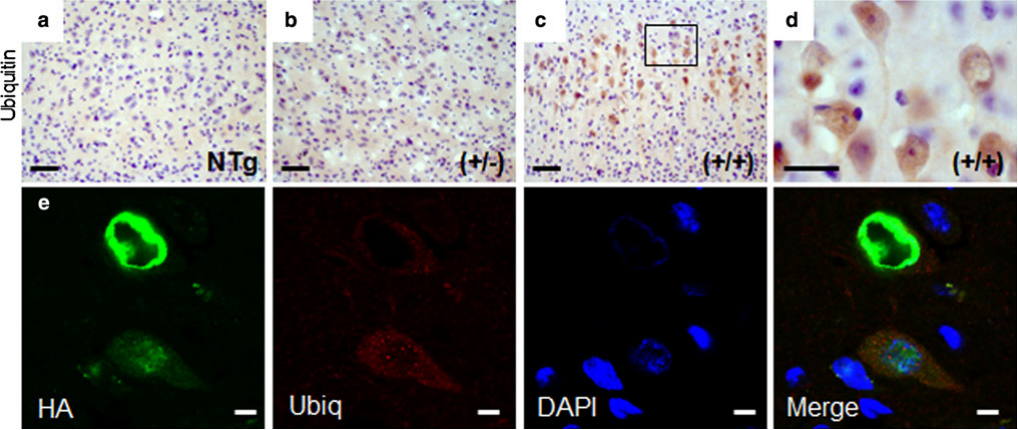
FUS staining does not colocalise with ubiquitin in the cortex. **a**–**c** Ubiquitin positive neurons were increased in the motor cortex of hFUS (+/+) (**c**) and to a much lesser extent (+/−) (**b**) animals compared to NTg controls (**a**) (*scale bar* 50 μm), and **d** weak ubiquitin staining is prevalent in hFUS (+/+) mice (*scale bar* 20 μm). **e** Fluorescent labelling of HA-tagged FUS and ubiquitin in the motor cortex of a hFUS (+/+) mouse shows cytoplasmic FUS deposits surrounded by ubiquitin, and diffuse granular cytoplasmic Fus staining accompanied by small ubiquitin positive cytoplasmic granules but no co-labelling of the two proteins (*scale bar* 5 μm)

Ubiquitin staining in the brain revealed an increase in faint diffuse, ring-like staining in the motor and somatosensory cortices of hFUS (+/+) mice, together with some more intensely stained ovoid structures in a subset of cells (Fig. 5c, d) while some minor staining was also observed in hFUS (+/−) (Fig. 5b) animals compared to their NTg littermates (Fig. 5a). There was no significant ubiquitin staining present in other brain regions. There was no obvious colocalisation between hFUS and ubiquitin staining in the cortex in hFUS (+/+) mice (Fig. 5e), rather, some neurons showed ring-like FUS structures with the ubiquitin staining appearing to encircle these concentrically, while other cells displayed diffuse cytoplasmic FUS staining, with small ubiquitin foci, and no apparent colocalisation between the two proteins. The identity of the more intensely stained ubiquitin-positive structures observed in some cells in the cortex is not currently clear, however, they do not appear to display any obvious colocalisation with Fus.

There was no sign of neuronal loss and markers for astroglial (GFAP) and microglial (CD68) reactivity were negative throughout the brain providing no evidence of an inflammatory reaction to a more subtle neurodegenerative processes in hFUS (+/−) or (+/+) mice up to 12 weeks (Online resource Fig S1). Given the recent report that FTLD-FUS is distinguished from ALS-FUS by the presence of Ewing’s Sarcoma protein (EWS) and TATA-binding protein-associated factor 15 (TAF15) colocalising with FUS pathology [41], we also looked for the presence of these proteins in our mice, but found no evidence of pathology or colocalisation of FUS with either protein (Online resource Fig S2).

### Overexpression of human wild-type FUS results ALS-like pathology in the spinal cord

**Fig. 6:**
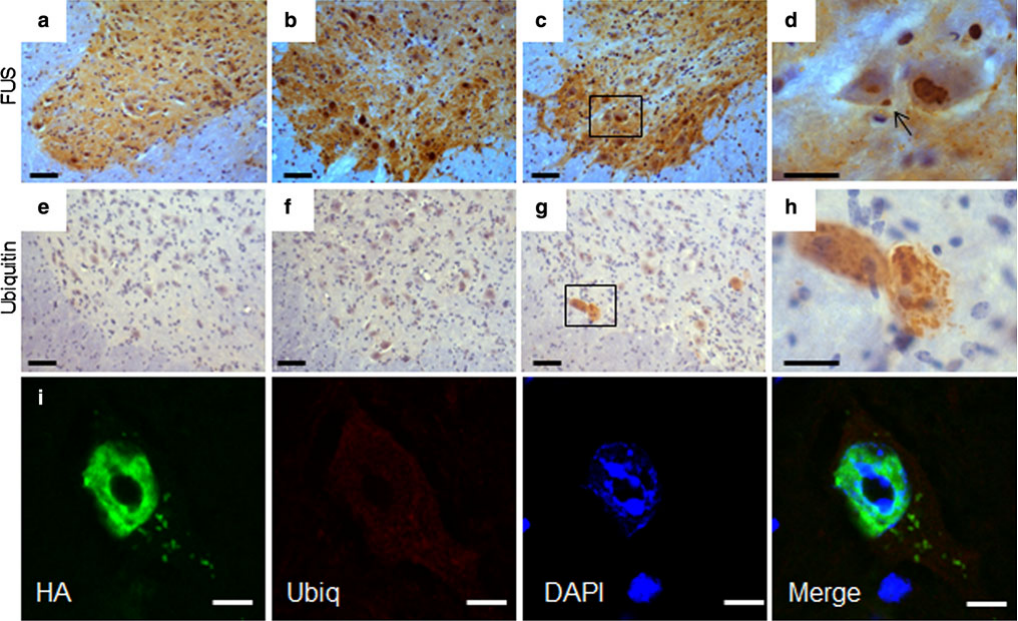
FUS and ubiquitin spinal cord pathology in hFUS mice. **a**–**c** FUS expression in the anterior horn of the spinal cord was increased in both (+/−) (**b**) and (+/+) (**c**) hFUS mice compared to their NTg littermates (**a**) (*scale bar* 50 μm). **d** A higher power image showing the presence of a FUS inclusion (*arrow*) in a large neuron of a hFUS (+/+) animal. (*scale bar* 20 μm). There was no evidence of inclusions in either hFUS (+/−) or NTg animals. **e**–**g** Abnormal granular ubiquitin was only present in a few large neurons of hFUS (+/+) (**g**) animals, with no significant staining in the anterior horn of the spinal cord of either NTg (**e**) or hFUS (+/−) (**f**) animals (*scale bar* 50 μm). **h** A higher power image showing rather diffuse, granular ubiquitin staining throughout two large neurons, with no obvious large inclusions visible (*scale bar* 20 μm). **i** Fluorescent labelling of HA-tagged FUS and ubiquitin in a neuron of the anterior horn of the spinal cord of a hFUS (+/+) mouse shows multiple small FUS inclusions in the cytoplasm of the cell with diffuse, low-level ubiquitin staining throughout the cytoplasm, but no evidence of HA-FUS and ubiqitin colocalisation (*scale bar* 5 μm)

In the spinal cord of hFUS (+/+) mice, FUS staining showed globular and granular cytoplasmic inclusions similar to those seen in human FUS-ALS patients [28, 55] (Fig. 6c, d), which were absent in hFUS (+/−) and NTg animals (Fig. 6a, b). Ubiquitin immunohistochemistry revealed occasional neurons in hFUS (+/+) mice with granular ubiquitinated deposits (Fig. 6g, h) but there was no colocalisation with FUS inclusions (Fig. 6i), which is similar to the pathology observed in FUS-ALS cases [55].

**Fig. 7:**
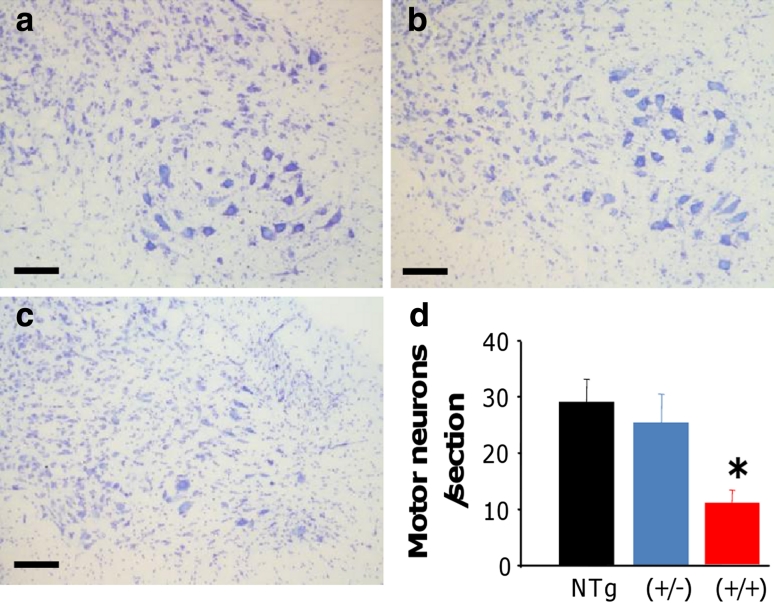
hFUS overexpression results in a loss of motor neurons in homozygous animals. **a**–**c** Cresyl violet staining of motor neurons in the lumbar ventral horn of NTg (**a**), hFUS (+/−) (**b**) and hFUS (+/+) (**c**) mice, showing a dearth of neurons present only in the hFUS (+/+) animals (*scale bar* 100 μm). **d** Cell counting of motor neurons in the lumbar anterior horn showed a significant loss of approximately 60 % of motor neurons in hFUS (+/+) mice compared with non-transgenic controls. In contrast hFUS (+/−) mouse motor neuron numbers were not significantly different (* *p* < 0.05)

**Fig. 8:**
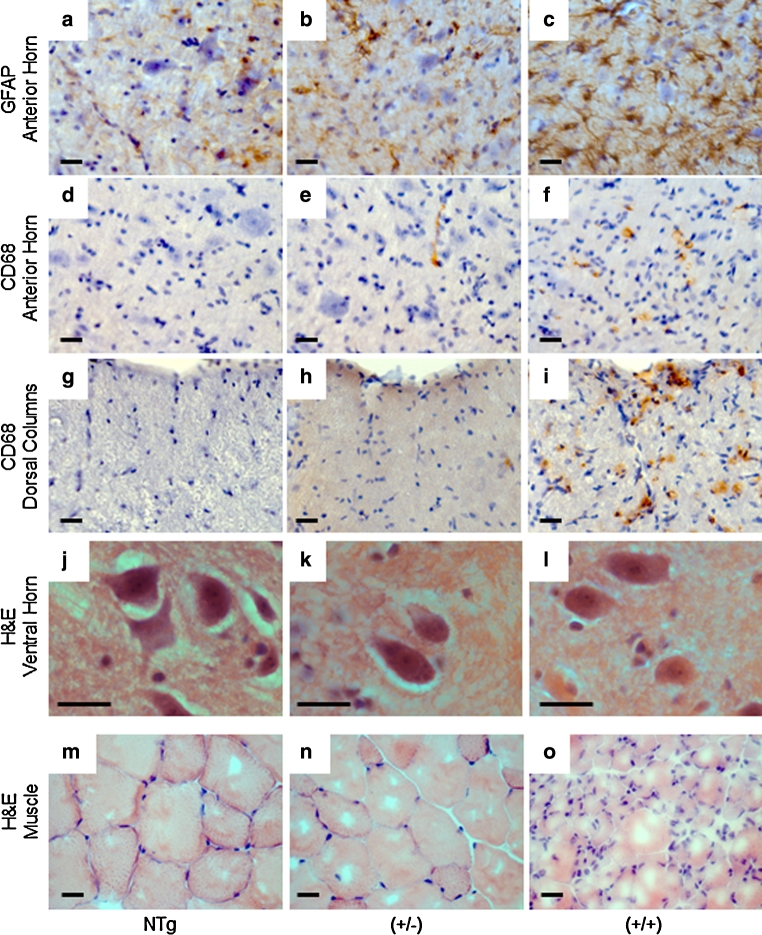
Increased neuroinflammation in the cord of hFUS mice accompanied by muscle atrophy. **a**–**c** Significant FUS expression-dependent increases in astrogliosis, detected using GFAP immunohistochemistry, were apparent in the anterior horn of the spinal cord in both (+/−) and (+/+) hFUS mice (*scale bar* 20 μm). **d**–**i** FUS expression-dependent increases in microglial activation, detected using CD68 immunohistochemistry, were apparent to a mild extent in the anterior horn of the spinal cord, (**d**–**f**) and more dramatically in the dorsal columns (**g**, **h**) of hFUS (+/+) mice. A very few cells were also present in hFUS (+/−) animals (*arrows*) (*scale bar* 20 μm). **j**–**l** Haematoxylin and eosin staining of spinal cord shows no evidence of eosinophilic inclusions in surviving motor neurons of end-stage hFUS (+/+) mice. **m**–**o** Haematoxylin and eosin staining of muscle showin*g* scattered and grouped muscle fibre atrophy, characteristic of motor neuron degeneration in hFUS (+/+) mice, with some evidence of muscle fibre disorganisation in hFUS (+/−) animals (*scale bar* 20 μm)

Neuronal cell counting demonstrated a significant (~60 %) loss of α-motor neurons in the anterior horn of the lumbar spinal cord in hFUS (+/+) animals compared to their hFUS (+/−) (*p* = 0.034) and NTg (*p* = 0.012) littermates (Fig. 7). This was associated with an increase in astrogliosis in the anterior horn (Fig. 8c), and to a lesser extent microgliosis in both the anterior horn (Fig. 8f) and the white matter tracts, in particular the dorsal columns (Fig. 8i). Haematoxylin and eosin staining of end-stage spinal cord tissues reveals no evidence of eosiniphilic aggregates in the cell bodies of surviving motor neurons in hFUS (+/+) mice compared to their NTg and hFUS (+/−) littermates (Fig. 8j, l), differing from reports in mice overexpressing wild-type TDP-34 [49], where these aggregates are proposed to be abnormal accumulations of mitochondia. Our finding suggests that Fus and TDP-43 may have differential effects on mitochondrial distribution, and hence their involvement in ALS may be via distinct mechanisms, although given the significant loss of motor neurons in our hFUS (+/+) mice we cannot rule out mitochondrial mislocalisation in the lost cells as a disease factor.

Muscle histology from end-stage hFUS (+/+) mice showed marked grouped atrophy of muscle fibres characteristic of denervation seen in muscle from ALS patients (Fig. 8o). Interestingly, age-matched hFUS (+/−) animals showed a milder disorganisation of their muscle fibres (Fig. 8n) compared to their NTg littermates (Fig. 8m). There was also a modest increase in the number of reactive astroglia (Fig. 8b) and the occasional activated microglial cell seen in the white matter tracts and anterior horn of hFUS (+/−) animals (Fig. 8e, h). Although neuronal counts were lower in hFUS (+/−) mice than in Ntg littermates these did not reach statistical significance (*p* = 0.53).

### Overexpression of wild-type FUS results in impaired neuromuscular function

Neuromuscular function was assessed in anaesthetised mice at 70 days of age. Maximum single twitch and tetanic force were determined in tibialis anterior (TA) and extensor digitorum longus (EDL) hind limb muscles.

Twitch and tetanic force recordings showed that the TA muscles in hFUS (+/+) mice were approximately 80 % weaker than in NTg (both *p* < 0.001) animals. In contrast, hFUS (+/−) TA twitch and tetanic force recordings were not significantly different from NTg animals indicating that there was no motor neuron dysfunction in these mice. A similar loss of muscle force was observed in hFUS (+/+) mice EDL compared to NTg mice, with a 20 % reduction in both twitch (*p* < 0.001) and tetanic force (*p* = 0.024; Online resource Fig. S3 and 9a, b).

**Fig. 9:**
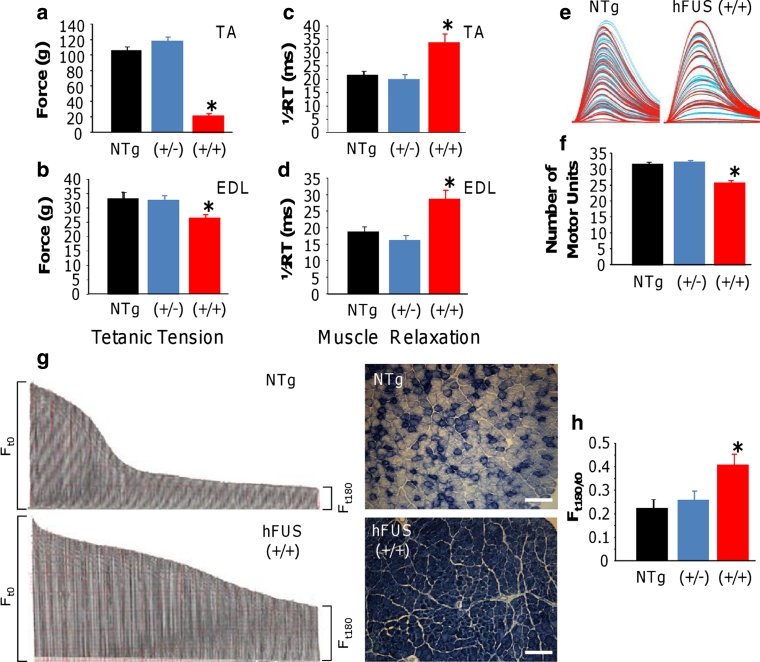
Neuromuscular function in hFUS mice. **a**, **b** Tetanic force production of the hind limb TA (**a**) and EDL (**b**) muscles was significantly reduced in hFUS (+/+) mice compared to NTg mice, with a deficit in force of approximately 80 and 20 % in the TA and EDL, respectively. **c**, **d** Muscle relaxation in both muscles was significantly slowed in hFUS (+/+) mice compared to both NTg and hFUS (+/−) animals. **e**, **f** Motor unit number was recorded from the EDL of all groups. Typical examples of motor unit traces from an NTg and hFUS (+/+) mice are shown in (**e**), and the results of mean motor unit survival are summarised in the *bar chart* (**f**) demonstrating a loss of 20 % of motor units in hFUS (+/+) mice. **g** Characteristic fatigue traces from a NTg and hFUS (+/+) mouse, generated by tetanic stimulation of the fast EDL muscle over 180 s. Each tetanic contraction is represented by a *single line* in the trace, and the length of the line is relative to the force produced. The length of the force trace at *F*_t180_ (time = 180 s) as a ratio of that at *F*_t0_ gives a fatigue index (FI). Wild-type mice lose a large proportion of force at the end of the stimulation period compared with the beginning of the trace, thus having a low FI. In contrast, in hFUS (+/+) mice the EDL is less fatigable, as indicated by the higher FI. On the *right of each trace* is a cross-section of TA muscle obtained from NTg and and hFUS (+/+) mice stained for SDH. NTg animals display a mix of darkly stained, oxidative type I fibres and lightly stained, less oxidative fast type II fibres. In contrast, hFUS (+/+) mice display mainly dark staining, suggesting a transformation of muscle fibres into a slower phenotype. **h** Summary of FI in EDL muscle of NTg and hFUS (+/−) and (+/+) mice.

Changes in the contractile characteristics of TA and EDL muscles of hFUS (+/+) mice were also observed. Muscle relaxation was significantly longer in both the TA and EDL of hFUS (+/+) mice compared to hFUS (+/−) and NTg mice (*p* = 0.005 and 0.006, respectively; Fig. 9c, d). In addition, alterations in the fatigue characteristics of TA and EDL muscles of hFUS (+/+) mice were observed. Fast-twitch muscles are usually very fatiguable and cannot maintain force when repetitively stimulated. A Fatigue Index (FI) was calculated for TA and EDL by expressing the force generated after a 3-min period of stimulation as a ratio of initial force produced, where a FI of 1.0 indicates that the muscle is resistant to fatigue. In the EDL of hFUS (+/+) mice, there was a clear increase in fatigue resistance, compared to hFUS (+/−) and NTg mice (*p* = 0.08). These findings indicate that there is a loss of fast-twitch muscle fibres in EDL of hFUS (+/+) mice (Fig. 9g, h). Cross sections of TA muscles were stained with succinate dehydrogenase (SDH), confirming a loss of glycolytic fast-twitch muscle fibres (Fig. 9g). Moreover, physiological assessment of functional motor unit survival revealed that 20 % fewer motor units survived in EDL muscle of hFUS (+/+) mice (Fig. 9e).

## Discussion

ALS and FTLD are two neurodegenerative disorders that fall within an overlapping clinical and pathological disease spectrum that includes the TDP-43 and FUS proteinopathies [31, 32, 40]. FUS and TDP-43 are structurally related RNA-binding proteins that may have overlapping functions and form complexes with other RNA-binding proteins [25]. Both proteins reside predominantly in the nucleus, and shuttle between the nucleus and the cytoplasm to perform various functions [3, 14, 18, 36]. It has been proposed that both FUS and TDP-43 may induce disease via either a gain of function within the cytoplasm, or a loss of function from the nucleus. The loss of function hypothesis is supported by evidence of nuclear depletion of TDP-43 in neurons containing cytoplasmic inclusions [2, 12, 45], a finding that has not yet been conclusively reported for FUS.

FUS accumulates within cytoplasmic inclusions in ALS patients (and rarely FTLD cases) when it is associated with *FUS* gene mutations [28, 54, 55]. FUS containing inclusions have also been identified as the dominant pathology in a subset of FTLD patients accounting for most cases that have TDP-43 and tau negative pathology [38, 43, 48, 53]. We generated hemizygous and subsequently homozygous FUS transgenic mouse lines expressing HA-tagged human wild-type FUS at 1.4- and 1.9-fold above endogenous levels in non-transgenic littermates (respectively). Overexpression of human FUS resulted in significantly decreased expression of mouse Fus. The overall pattern of Fus positive staining in all animals, regardless of genotype, was consistent with that previously reported [1], with intense neuropil staining in the cord and to a lesser extent the brainstem, together with nuclear staining throughout the CNS, although neuropil staining throughout the brain appears slightly increased in hFUS (+/+) mice compared to their NTg and (+/−) littermates, which is consistent with the increase in cytoplasmic Fus observed in these mice.

Although additional attempts were made, we were unable to generate further viable transgenic lines over-expressing human wild-type FUS under the control of the Prion promoter for comparison to the line reported here. This is similar to problems described in TDP-43 rodent models of ALS [56, 59], and is likely to be due to selective pressure against the expression of FUS above endogenous levels during early development, given its apparent dose-dependent toxicity in cellular models [7, 16, 23].

Homozygous transgenic mice developed a rapidly progressive motor deficit as measured by rotarod, locomotor activity and neurophysiological testing. Tremor began around 4 weeks and progressed to paralysis at 10–12 weeks, necessitating euthanasia. End-stage pathology revealed approximately 60 % motor neuron loss from the lumbar spinal cord. Many surviving neurons contained FUS-positive, ubiquitin-negative inclusions in the spinal cord, and there was evidence of microglial and astrocytic activation in the anterior horn and white matter of the dorsal columns. These changes are the pathological hallmark of human mutant FUS-mediated ALS cases [55]. Most ALS-related FUS mutations reside in the C-terminal nuclear localising signal (NLS). They disrupt binding to the nuclear transport factor, transportin, which impairs their nuclear import, leading to cytoplasmic accumulation within motor neurons and toxicty [13]. The degree of cytoplasmic mislocalisation in cellular studies for each ALS mutant appears to correlate with the age of disease onset in ALS patients implying that cytoplasmic mislocalisation is directly toxic [13]. Conversely, deletion of the nuclear export signal strongly suppressed mutant FUS toxicity in *Drosophila*, implying that cytoplasmic localisation is required for neurodegeneration [29]. This finding is supported by a recent study in *C. elegans* demonstrating that cytoplasmic mislocalisation of FUS induced by mutant FUS is sufficient to cause motor dysfunction, even in the presence of functional levels of wild-type FUS in the nucleus [39]. Here, we show that overexpression of wild-type FUS is toxic to motor neurons when it accumulates in the cytoplasm. Thus, the toxicity of FUS mutations may be solely due to their impact on nuclear import [6, 13, 17, 22, 26]. Although nuclear Fus levels also increase in these animals, it is unlikely that this is responsible for the main pathological phenotype, as hemizygous animals show a significant increase in nuclear Fus, and yet show no signs of overt phenotype after two years of age. In the brain, there was no evidence of microglial and astrocytic activation or neuronal loss despite abundant granular and skein-like FUS-positive inclusions in the cytoplasm of multiple neuronal populations. Given the early onset severe motor dysfunction in these animals, it was not possible to assess them for cognitive impairment. In contrast, hemizygous animals have no gross pathological changes in either the brain or spinal cord at 12 weeks and show no evidence of motor dysfunction out to 104 weeks. They did however, show mild astroglial and microglial activation in the spinal cord at 12 weeks. There were also subtle changes within muscle architecture in hemizygous mice, without signs of neuronal loss or neurophysiological evidence of neuromuscular dysfunction. A cohort of hemizygous mice is currently being aged and will be screened for signs of cognitive dysfunction. Recent findings in FUS transgenic rats overexpressing wild-type FUS revealed cognitive defects in aged animals [21]. Fus overexpression within the CNS of our mice is variable, with some cell populations displaying more expression than others. This suggests that the PrP-driven expression may display some mosaic expression properties in this model. However, the lack of obvious cell loss in several brain cell populations showing high levels of Fus overexpression supports the idea that lower motor neurons are selectively vulnerable to Fus overexpression. We therefore conclude that the overexpression of human wild-type FUS is particularly toxic to motor neurons.

These findings are consistent with recent reports of wild-type FUS toxicity in other species. Overexpression of wild-type Fus results in punctate cytoplasmic aggregates and dose-dependent toxicity in yeast [16, 23] and apoptotic cell death in human prostate cancer cells [7]. Overexpression of human wild-type FUS restricted to neurons in *Drosophila* results in a dose-dependent decrease in life span [37], and an impaired locomotor phenotype accompanied by morphological abnormalities in motor neurons and neuromuscular junctions [8].

Overexpression of the R521C ALS FUS mutant in the rat [21] resulted in an aggressive motor phenotype leading to death of all animals by 10 weeks. This was accompanied by disruption of the neuromuscular junction and a ~10 % motor neuron loss. They report some FUS mislocalisation to the cytoplasm without discrete inclusion formation and although ubiquitinated inclusions were observed within spinal neurons they do not colocalise with FUS. Rats transgenically overexpressing wild-type human FUS have no motor phenotype or spinal cord pathology but develop cognitive dysfunction and neuron loss in the frontal cortex and dentate gyrus by 12 months. Here again, FUS localised to the nucleus with little cytoplasmic mislocalisation and no inclusions. Ubiquitinated inclusions were detected in FUS expressing cells but did not colocalise with FUS. Our transgenic mice display a progressive and lethal motor phenotype similar to that seen in the rats expressing mutant FUS. In our mice, neuronal loss was only seen in homozygous animals where significant cytoplasmic mislocalisation of FUS was observed without loss of nuclear FUS. Both mouse and rat transgenic models support a toxic gain of function due to the accumulation of FUS rather than a loss of nuclear function.

Our mice constitutively overexpress wild-type human FUS under the control of the prion promoter. In comparison, the transgenic rat lines, wild-type and mutant, employed a cDNA construct driven by the Tet-off TRE and tTA promoter system whereby expression is induced only on weaning [21]. Thus, it is possible that the different promoter systems and onset of expression, combined with species and strain variations, may account for some of the different specific effects that overexpression of the wild-type FUS has in these two models. Nevertheless, neurodegeneration was observed in both our wild-type FUS mice, and the wild-type FUS transgenic rats, demonstrating that FUS overexpression is pathogenic.

The phenotype observed in our hFus (+/+) mice bears some similarities to mice overepxressing wild-type TDP-43 [57], such as a failure to gain weight, tremor, and motor dysfunction. Since both Fus and TDP-43 are RNA-binding proteins, the similarities in dysfunction that occur in response to overexpression of either protein may suggest that dysfunctions in RNA processing are important in the development of disease.

We conclude that overexpression of wild-type human FUS will induce motor neuron degeneration in mice when protein levels are sufficient to cause significant cytoplasmic accumulation. Our mice reproduce many aspects of the clinical phenotype and pathological features of human mutant FUS-mediated ALS, and as such, this model will facilitate the exploration of disease mechanisms and opportunities for therapy.

## Electronic supplementary material

Below is the link to the electronic supplementary material.

Supplementary material 1. Movie S1. This video shows a representative example of the onset of tremor in a 4-week-old hFUS (+/+) mouse. (MPG 1985 kb)

Supplementary material 2. Movie S2. This video shows a representative example of the onset of mild hind limb dysfunction in the 4-week-old hFUS (+/+) mouse shown in Movie S1. This animal was still mobile. (MPG 1620 kb)

Supplementary material 3. Movie S3. This video shows a representative example of an increased hind limb paralysis in an 8-week-old hFUS (+/+) mouse, displaying a severely stilted gait, and an inability to raise the pelvis. (MPG 1638 kb)

Supplementary material 4 (PDF 418 kb)
