# Supplementary material for: Overexpression of human wild-type FUS causes progressive motor neuron degeneration in an age- and dose-dependent fashion
Source: Acta Neuropathol. 2012 Sep 9;125(2):273–88. doi: 10.1007/s00401-012-1043-z (PMC3549237; doi:10.1007/s00401-012-1043-z)
Supplement: Supplementary file 4 — Supplementary material 4 (PDF 418 kb) [file 401_2012_1043_MOESM4_ESM.pdf]

## Acta Neuropathologica

**Overexpression of human wild-type FUS causes progressive motor neuron degeneration in an age and dose-dependent fashion**

Jacqueline C Mitchell, Philip McGoldrick, Caroline Vance, Jemeen Sreedharan, Boris Rogelj, Elizabeth L. Tudor, Bradley N Smith, Christian Klasen, Christopher C J Miller, Jonathan D Cooper, Linda Greensmith, Christopher E Shaw†

†Corresponding author: christopher.shaw@kcl.ac.uk, King's Centre for Neurodegeneration Research, Kings College London, Departments Of Clinical Neurosciences[1] and [3]Neuroscience, Institute of Psychiatry, London SE5 8AF

## Supporting Information

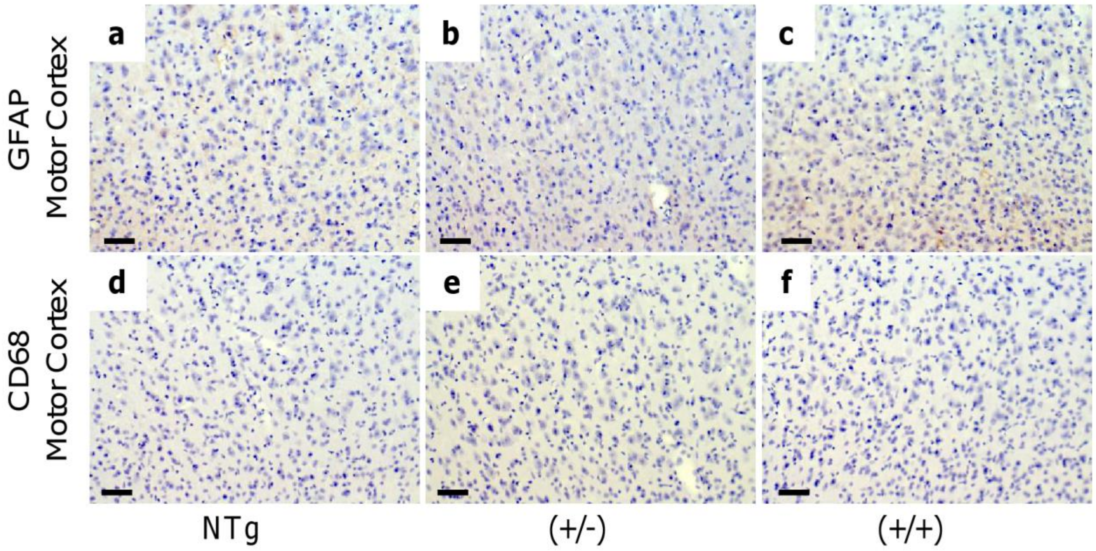


**Figure S1 hFUS overexpression does not alter neuroinflammation in the cortex.** (a-c) No changes in astrogliosis, detected using GFAP immunohistochemistry (d-f) or microgliosis, detected using CD68 immunohistochemistry, were detected in hFUS (+/-) or (+/+) animals (scale bar: 20µm)

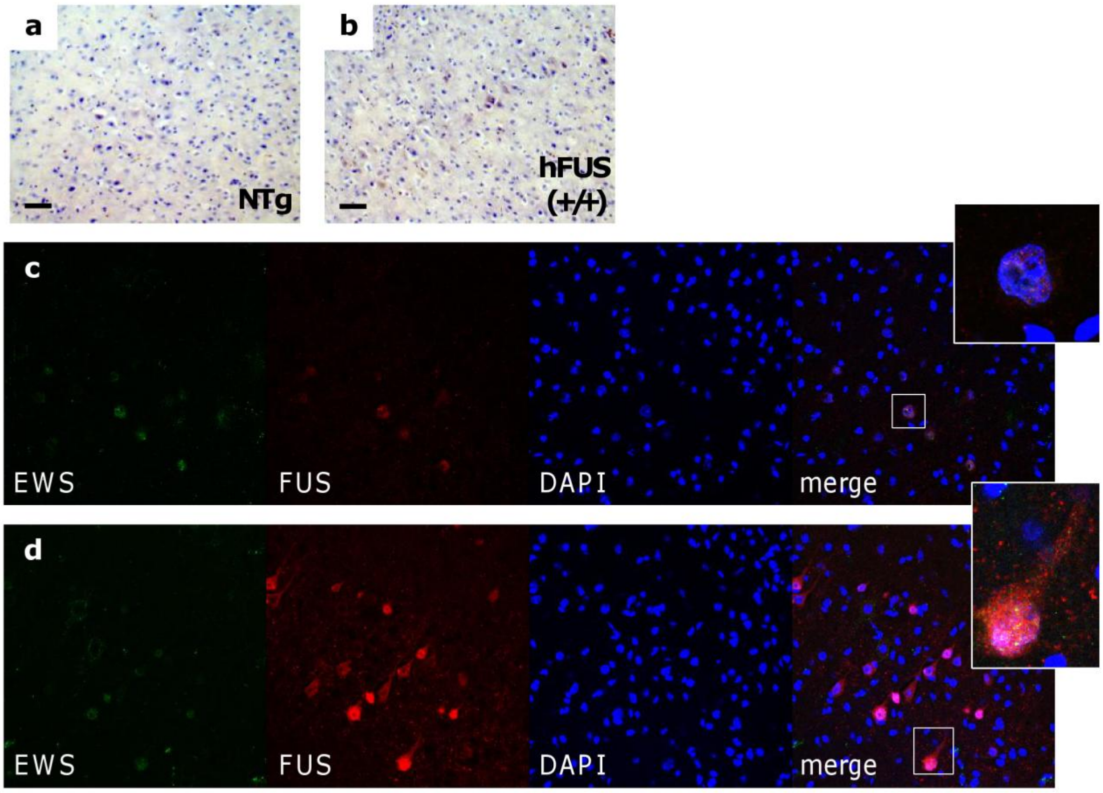


**Figure S2 FUS does not colocalise with EWS or TAF15 in the cortex of hFUS (+/+) mice.**

(a-b) No significant TAF15 staining was detected in NTg (A) or hFUS (+/+) (b) mice. (c-d) Low level EWS staining was detected in the nucleus of FUS positive cells in both NTg (c) and hFUS (+/+) (d) mice. However, total EWS levels did not appear to differ between NTg and hFUS (+/+) mice, and there was no evidence of FUS and EWS cytoplasmic colocalisation in hFUS (+/+) animals

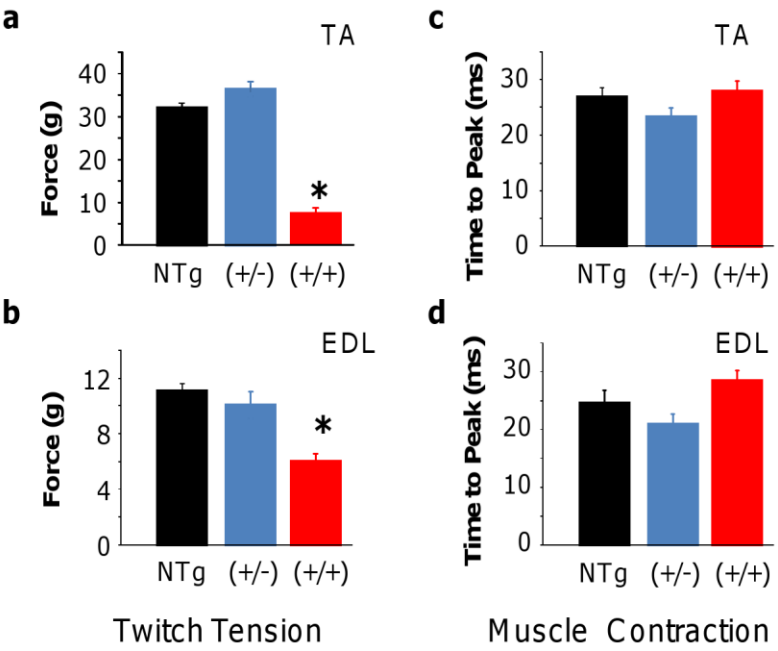


**Figure S3 Impaired twitch force but normal muscle contraction in hFUS homozygous mice.**

(a-b) Twitch force production of the hindlimb TA (A) and EDL (B) muscles was significantly reduced in hFUS (+/+) mice compared to NTg mice, with a deficit in force of approximately 80% and 20% in the TA and EDL respectively (*****$p<0.001$). (c-d) Muscle contraction measured as time to peak in both muscles was not significantly altered in response to hFUS overexpression
